# Comparison of the Shear Bond Strength of Orthodontic Composites Containing Silver and Amorphous Tricalcium Phosphate Nanoparticles: an *ex vivo* Study

**DOI:** 10.30476/dentjods.2022.94075.1760

**Published:** 2023-09

**Authors:** Zahra Tavakolinejad, Yasaman Mohammadi Kamalabadi, Arman Salehi

**Affiliations:** 1 Dept. of Orthodontics, School of Dentistry, Rafsanjan University of Medical Sciences, Rafsanjan, Iran; 2 General Dentist, School of Dentistry, Rafsanjan University of Medical Sciences, Rafsanjan, Iran; 3 Dept. of Restorative Dentistry, School of Dentistry, Rafsanjan University of Medical Sciences, Rafsanjan, Iran

**Keywords:** Nanoparticles, Orthodontic bracket, Orthodontic composite, Shear strength

## Abstract

**Statement of the Problem:**

It is important to use orthodontic composites with favorable properties, which are easily removed after the end of the treatment but not easily debonded during treatment. Nanoparticles have drawn attention for their antibacterial properties when added to composite resins. However, the effect of addition of nanoparticle on shear bond strength is not broadly discussed.

**Purpose:**

The present study was designed to compare the shear bond strength of orthodontic brackets bonded by orthodontic composite containing silver nanoparticles with orthodontic composite containing amorphous tricalcium phosphate nanoparticles.

**Materials and Method:**

In this *ex vivo* study, 36 sound extracted human premolars were used and randomly divided into three groups. The brackets were bonded in the first group by composite without nanoparticles, in the second group by composite containing 3% amorphous tricalcium phosphate nanoparticles and in the third group by composite containing 0.3% silver nanoparticles at the buccal surface of the teeth. The shear bond strengths of the samples were measured 24 hours after preparation by a universal testing machine. Data were analyzed using SPSS 21 software through one-way ANOVA and Tamhane's T2 multiple comparison tests. *p*Values under 0.05 were considered significant.

**Results:**

There was no significant difference between the mean shear bond strength of composite containing amorphous tricalcium phosphate nanoparticles with composite without nanoparticles (*p*= 0.142). However, the mean shear bond strength in the composite containing silver nanoparticles was significantly lower than the other two groups (*p*< 0.001).

**Conclusion:**

According to the results of this study, the addition of amorphous tricalcium phosphate nanoparticles to orthodontic composite does not significantly decrease the shear bond strength while silver nanoparticles reduce the shear bond strength of orthodontic composite.

## Introduction

To achieve the therapeutic goals of orthodontic treatment, it is important to have a firm bond between orthodontic brackets and dental enamel [ 1 ]. Nowadays, resin composite is widely used for this purpose by orthodontists because of its feasibility and shortened bracket bonding time [ 1 - 2 ]. It has been reported that among patients referring for orthodontic treatment, at least one patient complains about debonded brackets with an incidence of 0.6% to 28.3% [ 3 ]. On the other hand, brackets should be removed after treatment is finished. Therefore, the bond strength of the orthodontic brackets should with stand the forces applied during the treatment but not damage the tooth during the separation process [ 4 ]. The shear bond strength test is an evaluation procedure used for testing the adhesion of dental adhesives [ 5 ]. Many studies have shown that shear bond strength can be related to many factors such as type of etching material, types of brackets, bracket base design and size, adhesives, light-curing device, and restoration materials [ 6 - 8 ].

Orthodontic brackets, which are bonded to enamel, increase the risk of plaque accumulation and make their bonding sites difficult to be cleaned; both of which result in the demineralization of the enamel [ 9 ]. Several methods are used to decrease enamel demineralization in people having orthodontic appliances such as antibacterial mouth rinses and covering the brackets with agents inducing remineralization; however, they have not been promising [ 10 ]. If a suitable material, which can prevent the growth of bacteria in the area, is employed to attach the brackets, demineralization around the bracket might be reduced and subsequently the need for patient cooperation in oral hygiene is reduced [ 11 ]. The addition of nanoparticles to cement, orthodontic adhesives or acrylic resin can help in preventing microbial bonding and demineralization of enamel. Studies have shown that nanofillers reduce enamel demineralization without changing mechanical properties [ 12 - 14 ]. For instance, composites containing silver nanoparticles inhibit the growth of *Streptococcus mutans* and have a long-lasting antibacterial activity [ 15 ]. Furthermore, calcium ions (Ca^2+^) and phosphate ions (PO4^3-^) can remineralize decayed lesions. Composites containing calcium phosphate nanoparticles have been shown to induce remineralization, to have acid-neutralization properties, release calcium, and phosphor ions, especially in combination with poly (amido amine) [ 16 ].

Nanotechnology is the manipulation of matter at a specific dimension (1-100 nanometer), which results in remarkable features and applications [ 17 ]. Nanotechnology has been widely used in dental fields such as implants, radiography, periodontal diseases, restorative materials, and orthodontics [ 18 - 20 ]. Adding nanoparticles to composite resins can enhance their physical and mechanical features [ 20 ]. Many studies have shown the benefits of adding nanoparticles to composite resins regarding the antimicrobial properties and shear bond strength of orthodontic composite resins [ 21 - 23 ]. Lee *et al*. [ 24 ] revealed the antimicrobial activity of dental resin containing silver nanoparticles. Moreover, silver and hydroxyapatite nanoparticles in orthodontic adhesives were shown to improve the shear bond strength in a study conducted by Akhavan *et al*. [ 25 ]. Amorphous calcium phosphate nanoparticles added to orthodontic adhesive were also established to have antibacterial and remineralizing capabilities [ 26 ].

To the best of our knowledge, there is no study to examine the shear bond strength of orthodontic composites containing silver or amorphous tricalcium phosphate nanoparticles so far; hence, the present *ex vivo* study was designed to compare shear bond strength of orthodontic brackets bonded with orthodontic composite containing silver nanoparticles with orthodontic composite containing amorphous tricalcium phosphate nanoparticles. The null hypothesis was that the shear bond strength of composite containing nanoparticles is the same as those without nanoparticles.

## Materials and Method

This *ex vivo* study was performed on 36 sound human premolar teeth, which were extracted due to orthodontic treatment purposes. All teeth were free of any fluorosis, caries, cracks, abrasions, fractures, congenital and structural abnormalities, and there had been no chemical use (such as hydrogen peroxide) in the patients' dental history. Any debris and soft tissue debris were removed from samples, and all enamel surfaces were re-examined by a stereomicroscope (Nikon, Tokyo, SMZ 1500, Japan). The samples were kept in distilled water and at room temperature until the experiment [ 27 ].

The samples were cleaned with pumice powder by a rubber cup with a low-speed handpiece for 10 seconds, then washed, and dried with an oil-free air-water syringe handpiece [ 15 ]. Teeth were then mounted vertically in 2×2cm acrylic cylinders (Acropars, Karaj, Iran) in such a way that their crowns were exposed. They were randomly divided into three equal groups (n =12 in each group) based on the type of composite including Group 1: Transbond XT 3M Composite (3M, Unitek, St. Paul, MN, USA) without nanoparticle (control group), Group 2: Transbond XT 3M Composite (3M, Unitek) containing amorphous tricalcium phosphate nanoparticles, and Group 3: Transbond XT 3M Composite (3M, Unitek) containing silver nanoparticles.

To synthesize the nanoparticles of amorphous tricalcium phosphate (ATCP), one gram of hydroxyapatite (HA) was dissolved in 300 ml of deionized water and stirred vigorously to create a homogeneous suspension. Then drops of three Mol/L hydrochloric acid solutions (Merck, Darmstadt, Germany) were slowly (with a rate of 1 ml/min) added to the suspension, which completely dissolved the hydroxyapatite [ 29 ].

One Mol/L sodium hydroxide solution (Merck, Dar-mstadt, Germany) was added to the solution suddenly and rapidly, producing tricalcium phosphate. The pH of the solution was adjusted using ammonium solution (Merck, Darmstadt, Germany). The resulting precipitate was stirred vigorously for 5 minutes and separated from the solution by centrifuge (Binder, Tuttlingen, Germany). The gel-like deposit was washed three times, first with deionized water, then with ethanol-water solution (50-50 vol%) and finally with deionized water. Then the precipitates were transferred to petri dishes and 15ml of ethanol (Merck, Darmstadt, Germany) was added to it and after evaporation of ethanol, the sediment dried. Finally, the resulting deposit was placed at 80°C for one hour [ 28 - 29 ].

In order to synthesize silver nanoparticles (Ag), co-precipitation method was used. 90 milligrams of silver nitrate (Merck, Darmstadt, Germany) were dissolved in 500 milliliters of triply distilled water and heated to boiling water temperature. Then at the same time, quickly and suddenly the reducing and stabilizing agent was added to the solution and subjected to intense stirring for 24 hours. After filtration, the precipitate dried at 40°C for 4 hours [ 30 ]. Nanoparticles have been weighed with digital scales with an accuracy of 0.0001 grams (Acculab digital scales, Edgewood, NY, USA).

In order to prepare the nanocomposites, silver nanoparticles with an average diameter of 40-60 nm and weight percentage of 0.3%, nanoparticles of amorphous tricalcium phosphate with an average diameter of 40-60 nm and weight percentage of 3%, were used. These particles were mixed evenly with orthodontic light cure composite (3M Unitek TransbondTM XT, NY, USA) in a completely dark environment for 5 minutes by a high-speed mixer (3500 rpm) [ 11 , 31 ].

In order to prepare the samples, first, 37% phosphoric acid was used for 30 seconds in the shape of a 3×3 mm square on the buccal surface of the teeth, and then the teeth were washed with water for 10 seconds. The surface of the teeth was dried using an air syringe handpiece and after the white chalky surface appeared, Transbond XT 3M bonding (3M, Unitek, St. Paul, MN, USA) was applied on the teeth surface according to the manufacturer's instructions. They were then cured for 15 seconds using LED light-curing device (DEmetron A.2, kerr Italia, S.p.A. scafati, Italy) with a light intensity of 1000 mW/cm^2^ and a distance of one mm from the surface of each tooth [ 27 ].

Finally, stainless steel brackets (Ortho Organizer, CA, USA) were put in the center of the buccal surface of each tooth by a bracket holder (SIA, Casrta, Italy) with composites and after extra composite was removed by a dental explorer, each tooth was cured for 40 seconds [ 14 ].

Prepared samples were kept in distilled water at 37°C for 24 hours [ 32 ]. Then, the samples were subjected to thermal cycle operation 1000 times with a thermocycler (Vafaei Company, Tehran, Iran) between 5°C and 55°C for 15 seconds at each temperature with a transfer time of 10 seconds [ 13 ]. Finally, an Instron machine (Testometric M350-10, CT, England) with a speed of 0.5 mm/min and a 0.3 mm thick blade was used to measure shear bond strength. In this method, a shear force is applied parallel to the longitudinal axis of the tooth and the bracket, between the bracket and the tooth until the bracket is separated.

The shear bond strength (Mpa) was calculated by dividing the force applied to the composite cylinder (N) by the cross-sectional area of the specimens (πr^2^) [ 33 ]. Adhesive fracture patterns of samples were observed using a stereomicroscope (Nikon, Tokyo, SMZ 1500, Japan) with a magnification of 10× and by two trained individuals. Then the residual adhesive amount (Adhesive remnant index; ARI) were scored as (0) all composite resin remains on the bracket, (1) ≤50% of the resin composition remains on the tooth, (2) >50% of the resin composite remains on the tooth, and (3) all the resin composite remains on the tooth [ 34 ].

Eventually, the data on the shear bond strength and the fracture pattern of the composite in the studied groups were compared statistically. After compiling, the checklist information was entered into the SPSS statistical software version 21, respectively. The results were reported as "standard deviation ± mean" for quantitative data and as "(percentage) number" for qualitative data.

The Kolmogorov-Smirnov nonparametric test was used to assess the normality of the frequency distribution of shear bond strength in the studied groups (no nanoparticles, containing silver nanoparticles, containing amorphous tricalcium phosphate nanoparticles). Levene's test for homogeneity of variances was also used to evaluate the homogeneity of variance of shear bond strength in the studied groups. One-way ANOVA analysis was used to compare the mean of the shear bond strength (Mega Pascal) in the studied groups and Tamhane's T2 multiple comparison test was used to compare the mean of the shear bond strength (mega Pascal) in the studied pair groups.

The Kruskal-Wallis H test was also used in the study groups to compare the adhesive remnant index (at the tooth surface, inside the composite, and at the bracket surface). The significance level was set at 0.05.

## Results

The nonparametric Kolmogorov-Smirnov test showed that the frequency of shear bond strength of bonded brackets by orthodontic composite without nanoparticles (*p*= 0.526), orthodontic composite containing silver nanoparticles (*p*= 0.573) and orthodontic composite containing amorphous tricalcium phosphate nanoparticles (*p*= 0.510) had a normal distribution.

As shown in Table 1, One-way analysis of variance (ANOVA) showed that the difference between the average shear bond strength (MPa) of the three groups was statistically significant (*p*= 0.001).

**Table 1:** Comparison of average shear bond strength (MPa) in the three groups

Group	Composite without nanoparticles (n=12) Mean±Standard Deviation	Composite with silver nanoparticles (n=12) Mean±Standard Deviation	Composite with amorphous tricalcium phosphate nanoparticles (n=12) Mean±Standard Deviation	*P* Value
Variable				
Shear bond strength (MPa)	181.25±31.49	51.25±12.40	132.08±73.52	<0.001

Since Levene's test for assessing the homogeneity of variances of the shear bond strength in the three groups showed that the variance of shear bond strength in the studied groups was statistically significant (*p*= 0.001), Tamhane's T2 multiple comparison test was used to compare the mean shear bond strength in the pairs of the studied groups.

Tamhane's T2 multiple comparison test showed that the average shear bond strength of orthodontic composite containing silver nanoparticles was significantly lower than that of the orthodontic composite without nanoparticles (*p*<0.001). In addition, the average shear bond strength of the composite containing silver nanoparticles was significantly less in comparison with the orthodontic composite containing amorphous tricalcium phosphate nanoparticles (*p*= 0.005). However, the average shear bond strength of composites containing amorphous tricalcium phosphate nanoparticles did not show a statistically significant difference compared to composites without nanoparticles (*p*= 0.241).

The findings shown in Table 2 indicate that the fracture point of the composite during debonding process was favorable in the composite group containing silver nanoparticles due to its proximity to the bracket compared to the composite group without nanoparticles and the composite group containing nanoparticles of amorphous tricalcium phosphate. In addition, the fracture point was more favorable (closer to the bracket) in the composite group without nanoparticles than in the composite containing nanoparticles of amorphous tricalcium phosphate.

**Table 2:** Distribution of adhesive remnant index (ARI) in the three groups

Group	Composite without nanoparticles Frequency (percentage)	Composite with silver nanoparticles Frequency (percentage)	Composite with amorphous tricalcium phosphate nanoparticles Frequency (percentage)
Variable			
All the resin composite remains on the bracket	2 (16.7%)	0 (0%)	4 (33.3%)
Less than 50% of the resin composite remains on the tooth	2 (16.7%)	3 (25%)	2 (16.7%)
More than 50% of the resin composite remains on the tooth	6 (50%)	4 (33.3%)	6 (50%)
All the resin composite remains on the tooth	2 (16.7%)	5 (41.7%)	0 (0%)
Total	12 (100%)	12 (100%)	12 (100%)

Since the adhesive remnant index (ARI) is defined as a qualitative ordinal variable (0, 1, 2 and 3), in order to compare the median ARI in the three groups, the nonparametric Kruskal-Wallis H test was used. The results of this test showed that the median ARI in the three groups was not statistically significant (*p*= 0.054). Table 2 shows the frequency (percentage) of the composite failure pattern in the three groups.

## Discussion

Different nanoparticles can be added to orthodontic composite to enhance the antibacterial properties of the composite such as silver and amorphous calcium phosphate [ 35 ]. Silver nanoparticles are shown to inhibit biofilm viability and growth, especially *Streptococcus mutans* [ 15 ]. The antibacterial mechanism of silver is not fully understood yet; however, it is suggested that the active oxygen produced by the action of light energy in presence of silver as a catalyzer can damage the structure of bacteria [ 36 ]. Amorphous calcium phosphate nanoparticles also have the properties of moderately reducing *Streptococcus mutans* growth and acid neutralizing by releasing calcium and phosphate ions [ 37 ]. 

In addition to antibacterial properties, it is essential that the nanoparticles added to the composite maintain a reasonable shear bond strength or in a more favorable condition, to improve this property. In this study, the effect of adding concentrations of 0.3% silver nanoparticles and 3% amorphous tricalcium phosphate nanoparticles to composite on the shear bond strength of orthodontic brackets were evaluated. The logic behind choosing these nanoparticles for the purpose of this study is that the antibacterial properties and shear bond strength of orthodontic adhesives containing these nanoparticles have been verified in some studies [ 26 , 38 - 39 ]. Therefore, we conducted this study to investigate if adding these nanoparticles, silver and amorphous tricalcium phosphate, to the orthodontic composites has any influence on shear bond strength.

Our study indicated that the mean shear bond strength of composites containing silver nanoparticles was significantly lower than that of composites without nanoparticles, while the mean shear bond strength of composites containing amorphous tricalcium phosphate was not significantly different from composites without nanoparticles. During orthodontic treatment, reduction of shear bond strength leads to detachment of the bracket from the tooth surface and complicates the procedure [ 4 ]. On the other hand, the presence of silver nanoparticles causes discoloration in the composite, which is not aesthetically pleasant [ 21 ]. Conversely, the shear bond strength of composites containing amorphous tricalcium phosphate nanoparticles is virtually similar to a composite without nanoparticles and has the ability to release phosphorus and calcium ions during treatment, which can help the process of remineralization of decayed lesions. Moreover, it raises the pH of the environment from 4 to 6.5 which helps neutralize bacterial acids and reduce decay [ 15 , 40 ].

In the study of Eslamian *et al*. [ 39 ], the same size (50 nm) and weight concentration (0.3%) of silver nanoparticles as the present study was added to orthodontic adhesives; the results were also consistent with the present study as they revealed that silver nanoparticles decreased the shear bond strength of orthodontic adhesives. In another study, examining the shear bond strength of adhesives containing nanoparticles of silver, zinc oxide, and titanium oxide, Reddy *et al*. [ 27 ] found that adhesives containing nanoparticles had lower shear bond strength compared to orthodontic adhesives without nanoparticles. The results of their study were similar to the present study in the composite group containing silver nanoparticles as the shear bond strength in this group was reduced compared to the control group. In addition, more than 0.33% reduction was shown in shear bond strength of orthodontic adhesives containing silver nanoparticles [ 14 ]. This is probably because the surface-area-to-volume ratio in nanoparticles is higher due to their smaller size, which results in more surface energy; thus, they tend to have more interaction with water and absorb moisture, and composite reactants react less due to the presence of water molecules. This is true for both silver nanoparticles and amorphous tricalcium phosphate nanoparticles, and since the size of silver nanoparticles is smaller than that of amorphous tricalcium phosphate nanoparticles, moisture absorption is higher and shear bond strength reduces further [ 27 , 41 ].

Ahn *et al*. [ 42 ] showed no significant difference between the shear bond strength of experimental composite adhesives containing silica nanofillers and silver nanoparticles and conventional adhesives, in this study none of the three different concentrations (0, 250, and 500ppm) of silver nanoparticles (diameter<5nm) chang-ed the shear bond strength significantly. They suggested that if used in a proper amount, silver nanoparticles might not have adverse effects on the physical properties of the adhesive. Degrazia *et al*. [ 38 ], who used <150 nm silver nanoparticles concluded that incorporation of these nanoparticles into orthodontic adhesives decreases shear bond strength in comparison to the control group; however, they did not observe any significant difference between different concentrations (0.11%, 0.18% and 0.33%). The results of the present study seem to be more similar to the study of Degrazia *et al*. [ 38 ], as in both studies a decrease in shear bond strength was observed by adding silver nanoparticles. This can be due to the size of particles and the concentration being closer to this study rather than to the study of Ahn *et al*. [ 42 ].

The study of Melo *et al*. [ 15 ] on the properties of adhesives containing silver and amorphous calcium phosphate showed that regarding shear bond strength, adding 0.1% silver nanoparticles to primer and adhesive did not change the bond strength while adhesives containing 10% amorphous calcium phosphate nanoparticles had higher bond strength compared to the control group. However, they showed that by increasing this percentage to 40%, the shear bond strength decreased [ 15 ]. The possible explanations for the different results compared to ours can be the significant difference in the size of nanoparticles (2.7nm silver nanoparticles and 116 nm amorphous calcium phosphate nanoparticles), the proportion added to the resin, and different techniques (spray-drying versus dissolution) for adding nanoparticles to the resin. Moreover, in their study [ 15 ], the properties of these nanoparticles were assessed when were added to adhesive and primer while in the current study, it was assessed when they were added to the composite.

The limitations of this study include the small sample size, which results in the possibility of random error to remain. Moreover, focusing on a single property of a few (here two) different types of nanoparticles makes it difficult to realize how beneficial using nanoparticles in orthodontics can be. Further studies are needed to evaluate the advantage of nanoparticle application in orthodontic composites regarding other properties rather than shear bond strength. 

## Conclusion

The results of this study showed that silver nanoparticles decreased the shear bond strength of orthodontic composites significantly. However, composites containing tricalcium phosphate nanoparticles did not show a significant change in shear bond strength compared to composites without nanoparticles.

## Acknowledgment

We would like to thank Rafsanjan University of Medical Sciences, Dr. Mohammad Sabet, Dr. Mahsa Falakian and Dr. Mina Arab Nadavi for technical and financial supports.

## Conflict of Interest

The authors of this manuscript certify that they have no conflict of interest.
